# Two Cases of Neuroendocrine Transformation of Prostate Carcinoma in the Era of Expanding Therapeutic Options: Do the Increased Number of Available Treatments Translate Into Improved Patient Outcomes?

**DOI:** 10.7759/cureus.99831

**Published:** 2025-12-22

**Authors:** Rita Aranha, Filipe Veiga, Rafael Marques, Joana Melo, Sofia A Oliveira

**Affiliations:** 1 Oncology, Unidade Local de Saúde Entre Douro e Vouga, Santa Maria da Feira, PRT

**Keywords:** androgen receptor pathway inhibitors, disease progression, neuroendocrine prostate cancer, poly(adp-ribose) polymerase inhibitors, prostatic neoplasms

## Abstract

Neuroendocrine prostate cancer is an aggressive and increasingly recognized resistance phenotype arising in advanced prostate adenocarcinoma, particularly after prolonged androgen receptor-directed therapy. We describe two patients with metastatic prostate cancer who developed rapid clinical progression accompanied by disproportionately low prostate-specific antigen (PSA) levels, ultimately revealing transformation to neuroendocrine carcinoma. These cases highlight the diagnostic challenges associated with this entity, the importance of obtaining new tissue for histologic confirmation when radiologic progression diverges from biochemical markers, and the limited effectiveness of current therapeutic options. Overall, these cases emphasize the critical need for improved clinical recognition and for continued research into targeted therapeutic approaches capable of addressing this highly aggressive phenotype.

## Introduction

Prostate cancer is the second most common malignancy among men worldwide, with approximately 1.5 million new cases diagnosed annually, surpassed only by lung cancer (excluding non-melanoma skin cancer) [1]. We now live in an era of rapidly expanding therapeutic options as allies for androgen deprivation therapy (ADT), including combinations such as ADT and androgen receptor pathway inhibitors (ARPIs), chemotherapy with ARPIs, and poly(ADP-ribose) polymerase (PARP) inhibitors combined with ARPIs, among others.

As a mechanism of therapeutic resistance, prostate cancer cells may undergo lineage plasticity, shifting away from an androgen-driven phenotype. This process is marked by downregulation of the androgen receptor and prostate-specific antigen (PSA), accompanied by increased expression of neuroendocrine markers such as neuron-specific enolase, chromogranin A, and synaptophysin, which are central to establishing a diagnosis of neuroendocrine prostate cancer (NEPC) [2]. The loss of androgen-receptor signalling ultimately results in resistance to conventional prostate cancer treatments [2].

NEPC comprises a broad spectrum of histologic subtypes, ranging from highly aggressive small-cell carcinoma to large-cell neuroendocrine carcinoma, as well as mixed adenocarcinoma-neuroendocrine variants. Small-cell carcinoma is characterized by high mitotic activity, scant cytoplasm, and marked proliferative indices, whereas large-cell disease demonstrates more abundant cytoplasm and prominent nucleoli but retains neuroendocrine differentiation. Mixed or intermediate phenotypes, in which adenocarcinoma coexists with neuroendocrine features, are increasingly recognized in treatment-emergent cases and may represent transitional stages within the continuum of lineage plasticity. These subtypes differ in morphology, proliferative index, and clinical behaviour, yet all share features of androgen receptor independence and aggressive tumour biology.

NEPC can arise either as a de novo entity or as a treatment-emergent phenotype following prolonged androgen receptor-targeted therapy. While de novo NEPC is rare, making up less than 1% of all prostate cancer cases, treatment-emergent transformation can occur in 15-20% of patients with advanced prostate cancer, although estimates vary depending on diagnostic criteria, biopsy practices, and clinical suspicion [3-5]. In clinical practice, histologic confirmation of NEPC is frequently obtained from metastatic sites rather than repeat prostate biopsy, as metastatic lesions are often more accessible, carry lower procedural morbidity, and better reflect the aggressive, androgen-independent characteristic of this subtype.

However, NEPC remains an outlier when it comes to new and targeted treatments. Therapeutic options are limited, and outcomes are generally poor. Owing to the rarity of this entity and the paucity of prospective data, most therapeutic strategies are extrapolated from the management of neuroendocrine tumours arising in other primary sites.

We describe two cases of advanced prostate adenocarcinoma that evolved into treatment-emergent neuroendocrine carcinoma, highlighting distinct clinical trajectories but similarly aggressive outcomes.

## Case presentation

Case 1

A 63-year-old man with an Eastern Cooperative Oncology Group (ECOG) Performance Status of 1 and a medical history of well-controlled hypertension, dyslipidaemia, and childhood poliomyelitis without residual deficits, presented with worsening nocturia. Laboratory evaluation revealed a markedly elevated prostate-specific antigen (PSA) level of 87 ng/mL. Prostate biopsy demonstrated adenocarcinoma of the prostate, Gleason score 7 (4+3), located in the right lobe.

**Figure 1:**
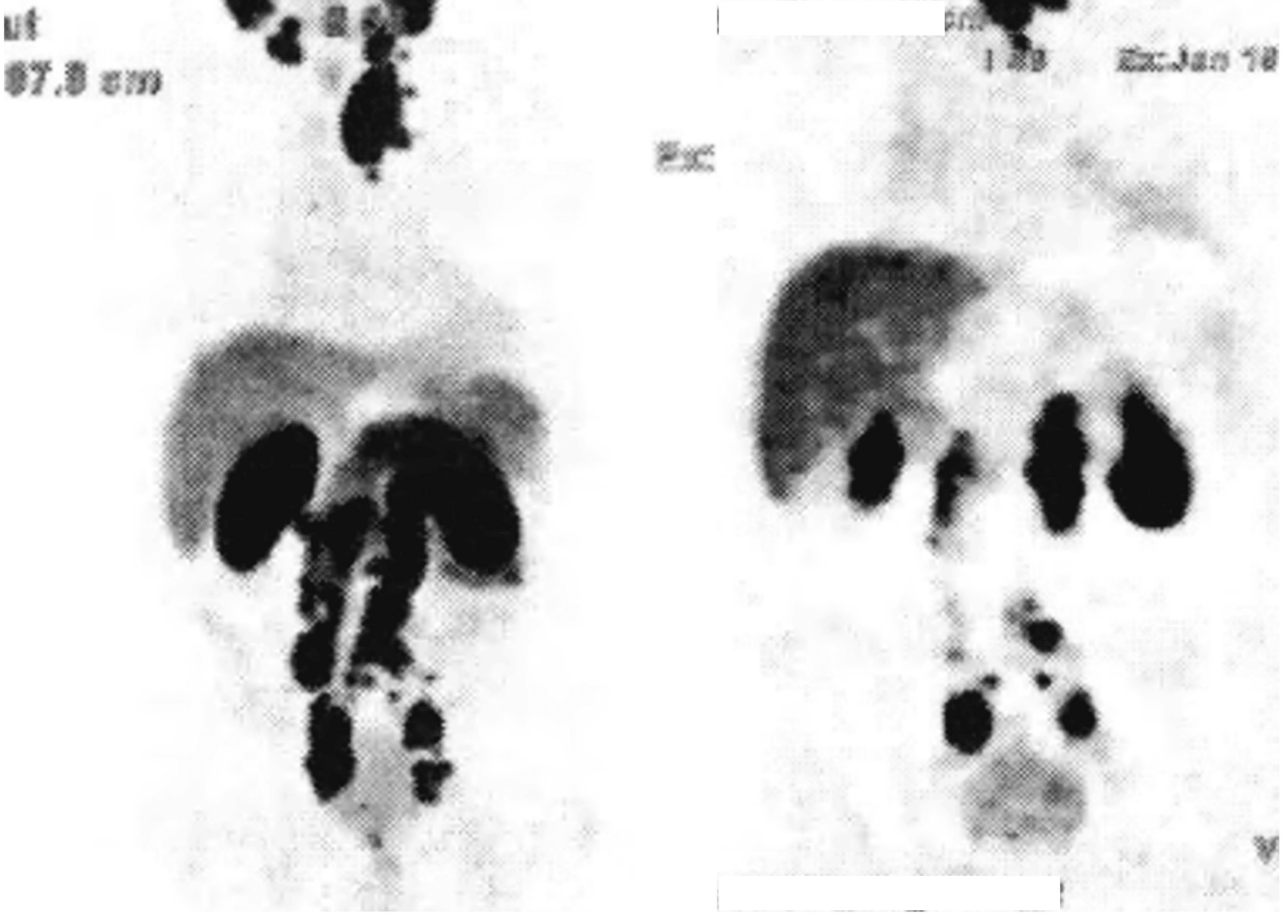
Staging PSMA PET scan showing extensive nodal metastatic involvement (Case 1) PSMA: prostate-specific membrane antigen; PET: positron emission tomography

Pelvic magnetic resonance imaging (MRI) showed a prostate lesion with capsular invasion, consistent with cT3 disease, along with suspicious regional lymphadenopathies. Staging body computed tomography (CT) scan confirmed multiple lumboaortic and pelvic lymphadenopathies. Bone scintigraphy showed no evidence of secondary lesions. A 68Ga-prostate-specific membrane antigen positron emission tomography (PSMA PET) scan revealed extensive nodal metastatic involvement with intense radiotracer uptake in the bilateral iliac, intercavo-aortic, retrocrural, mediastinal, and left laterocervical nodes, the latter forming an ill-defined nodal conglomerate (Figure 1). These findings were consistent with cT3aN1M1a prostate cancer.

The case was reviewed in a multidisciplinary tumour board where palliative systemic treatment was proposed. As the patient did not meet criteria for high-volume disease, first-line doublet therapy with goserelin and apalutamide was selected as the most appropriate approach in the hormone-sensitive setting. After four months, response assessment demonstrated a partial radiologic response in both the prostate and all previously involved lymph nodes, accompanied by a marked biochemical response, with the PSA level decreasing to 0.09 ng/mL.

**Figure 2:**
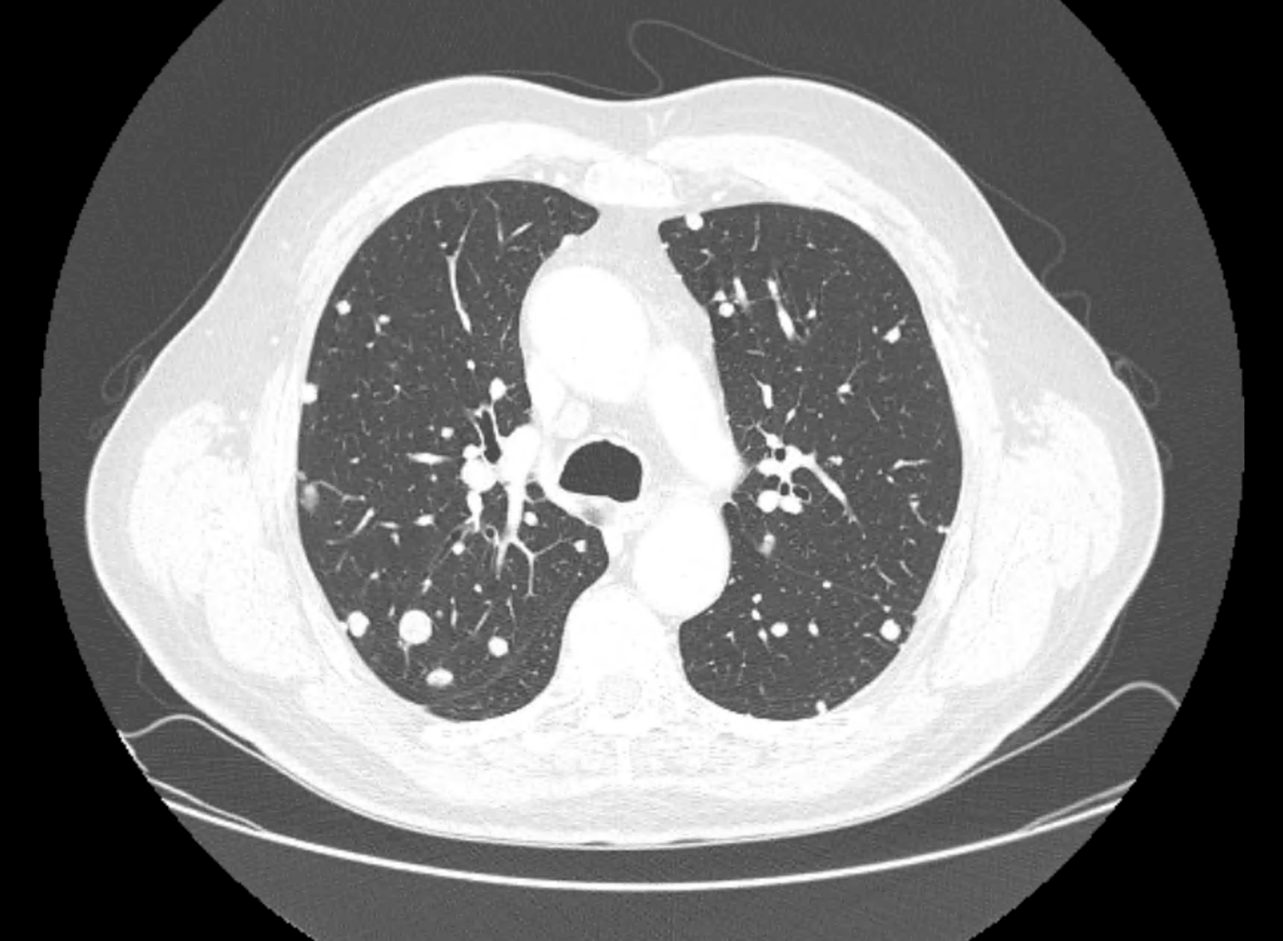
Follow-up chest CT after 10 months on apalutamide (Case 1) Extensive pulmonary metastases displaying a “balloon-release” pattern.

**Figure 3:**
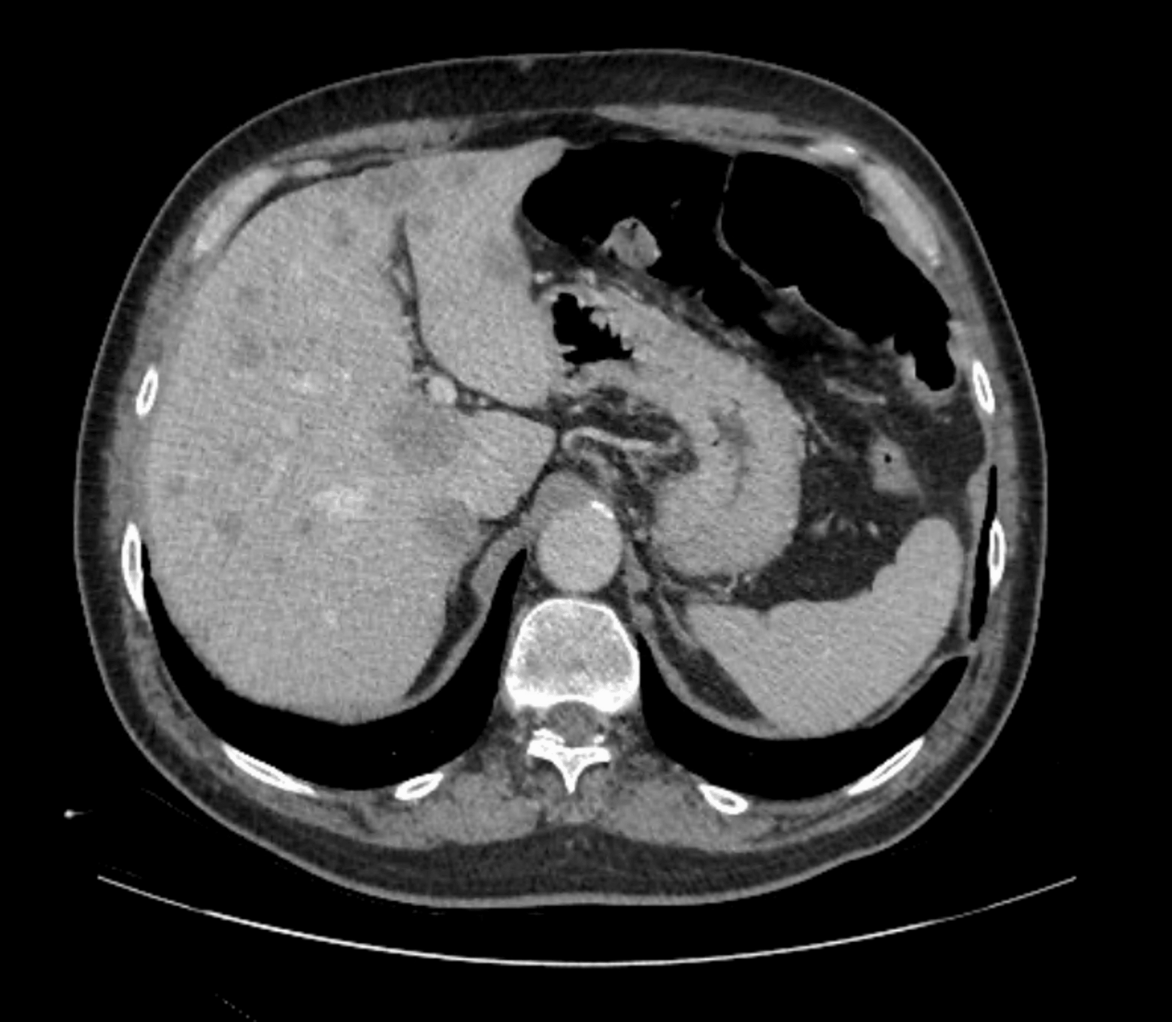
Follow-up abdominal CT after 10 months on apalutamide (Case 1) New metastatic hepatic lesions.

However, after 10 months of treatment, a body CT scan demonstrated disease progression, with enlargement of the supra and infraclavicular, mediastinal, and hilar lymph nodes, as well as the emergence of extensive pulmonary metastases displaying a “balloon-release” pattern (Figure 2). New hepatic metastases were also identified, along with progression of bone disease (Figure 3). Notably, at the time of radiologic progression, the PSA level remained 0.01 ng/mL.

Due to progressive worsening of urinary symptoms, the patient underwent a transurethral resection of the prostate (TURP), which revealed high-grade prostate adenocarcinoma, Gleason score 9 (5+4). Given the rapid clinical deterioration, second-line systemic therapy with docetaxel was promptly initiated.

Meanwhile, a liver biopsy identified metastatic small-cell neuroendocrine carcinoma, with a Ki-67 proliferation index exceeding 90%, and immunohistochemical markers suggesting prostatic origin. The case was subsequently re-evaluated in a multidisciplinary tumour board. In the absence of a dominant pulmonary lesion or any new imaging findings suggestive of an alternative primary neuroendocrine tumour, neuroendocrine transformation of prostate carcinoma was considered the most likely diagnosis.

**Figure 4:**
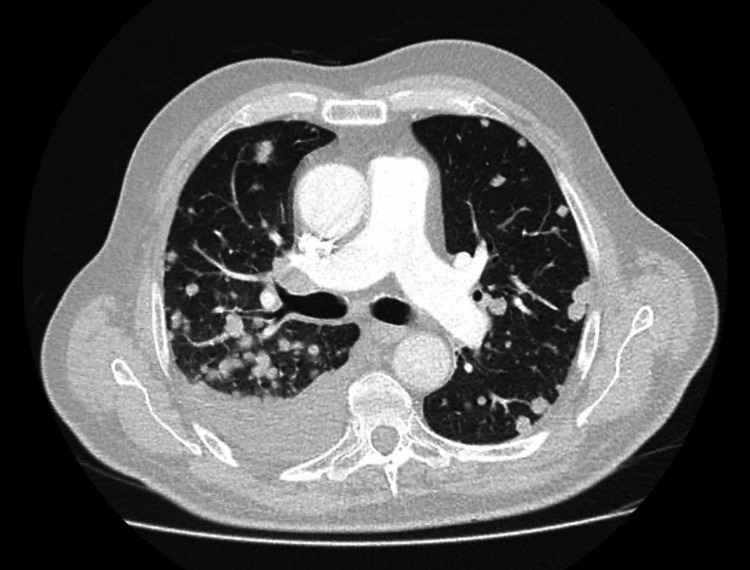
Follow-up chest CT after three cycles on docetaxel and carboplatin (Case 1) Progression of pulmonary metastatic lesions along with de novo pleural effusion.

**Figure 5:**
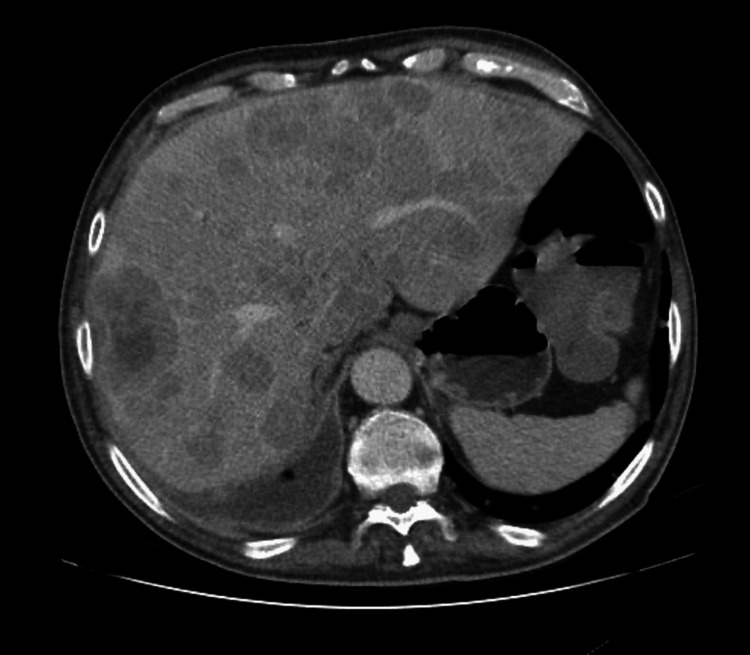
Follow-up abdominal CT after three cycles on docetaxel and carboplatin (Case 1) Progression of hepatic disease.

Carboplatin was added to ongoing docetaxel based on multidisciplinary assessment, as the patient had already initiated docetaxel before histologic confirmation of NEPC. Although platinum-etoposide is generally considered the preferred first-line regimen, a taxane-platinum combination was selected to avoid delaying treatment and is regarded as an acceptable alternative in selected clinical contexts. The patient completed three cycles of this combination. Restaging after these three cycles demonstrated progression of the primary prostate lesion, with further local extension and worsening nodal, pulmonary, and hepatic disease, along with the appearance of new pleural and peritoneal metastases (Figures 4, 5). Clinically, the patient reported increasing pain and worsening urinary dysfunction. Given the symptom burden at this time, the palliative care team was involved to assist in comprehensive symptom management.

During treatment, an expanded molecular analysis was performed using next-generation sequencing (NGS). In the absence of other actionable alterations and given the biological plausibility suggested in emerging data, the multidisciplinary team considered PARP inhibition as a potential therapeutic strategy, while acknowledging that the benefit of PARP inhibitors in cases of isolated MLH1 loss remains uncertain in NEPC. Based on the clinical course and molecular profile, third-line therapy with enzalutamide plus talazoparib was proposed; however, this regimen was not approved by higher regulatory bodies.

As an alternative, the patient commenced topotecan as third-line treatment. Despite treatment, the disease continued to progress, and the patient died after four cycles.

Case 2

A 65-year-old man with an ECOG Performance Status of 1 and a medical history of well-controlled hypertension, type 2 diabetes mellitus, dyslipidaemia, and a previous cholecystectomy was referred to Urology for evaluation of an asymptomatic yet markedly elevated PSA of 850 ng/mL. Digital rectal examination revealed induration of the right apical region. Prostate biopsy demonstrated high-grade adenocarcinoma, with a Gleason score of 9 (5+4) and a cribriform pattern in the right lobe, and a Gleason score of 10 (5+5) in the left lobe.

**Figure 6:**
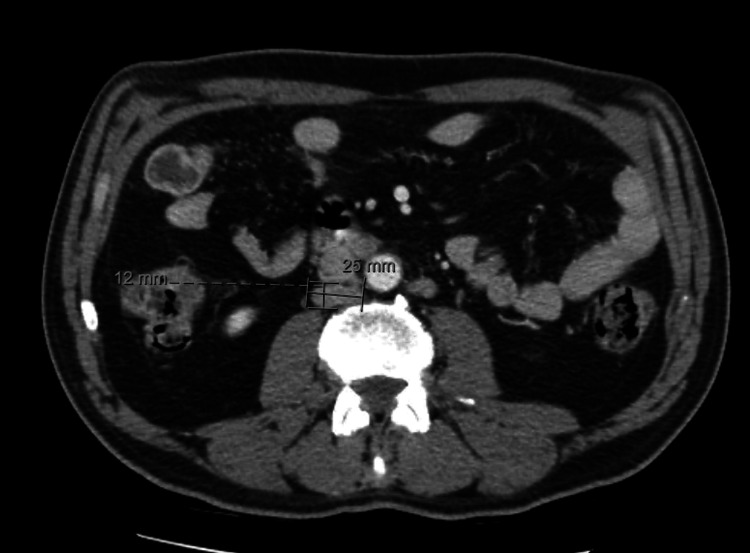
Staging CT scan showing multiple enlarged lumboaortic lymph nodes (Case 2) The largest measured 25 × 12 mm in the retrocaval region.

**Figure 7:**
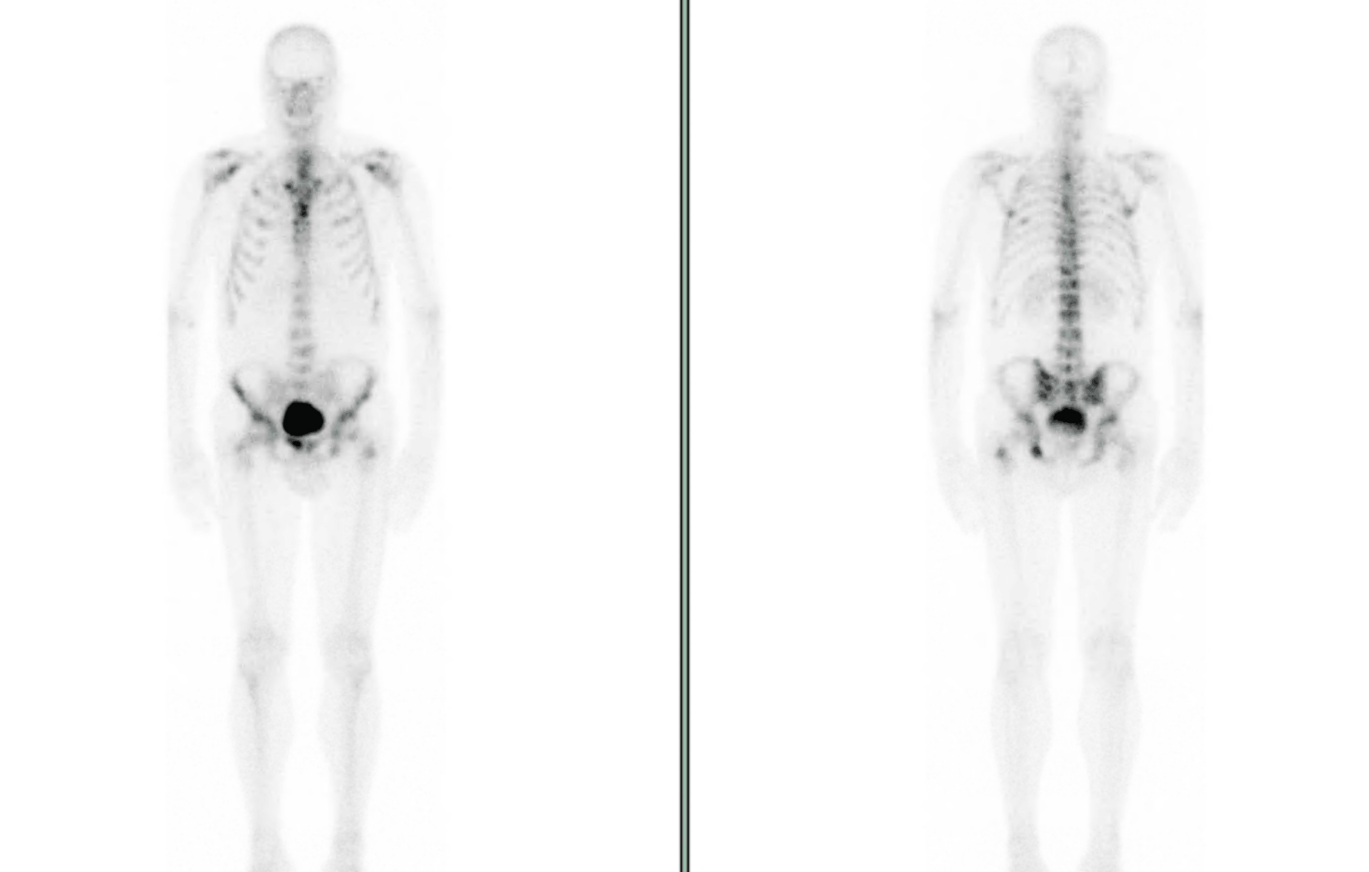
Staging bone scintigraphy (Case 2) Widespread osseous metastatic disease involving both the axial and appendicular skeleton.

Staging pelvic MRI showed seminal vesicle invasion, right external iliac lymph node involvement, and bone infiltration of the left ischiopubic ramus. Staging CT revealed multiple enlarged lumboaortic lymph nodes, extending from the renal hilum to the aortic bifurcation, the largest measuring 25 × 12 mm in the retrocaval region, as well as numerous pelvic lymphadenopathies (Figure 6). Bone scintigraphy demonstrated widespread osseous metastatic disease involving both the axial and appendicular skeleton (Figure 7). In sum, the data were consistent with cT3bN1M1c prostate cancer.

The case was discussed in a multidisciplinary tumour board, which recommended initiation of palliative systemic therapy. Given the high-volume disease, the patient began triplet systemic treatment with goserelin, docetaxel, and darolutamide. He completed six cycles of docetaxel, continuing thereafter on goserelin and darolutamide. Treatment was well tolerated, and a significant biochemical response was observed, with the PSA decreasing to 0.05 ng/mL.

**Figure 8:**
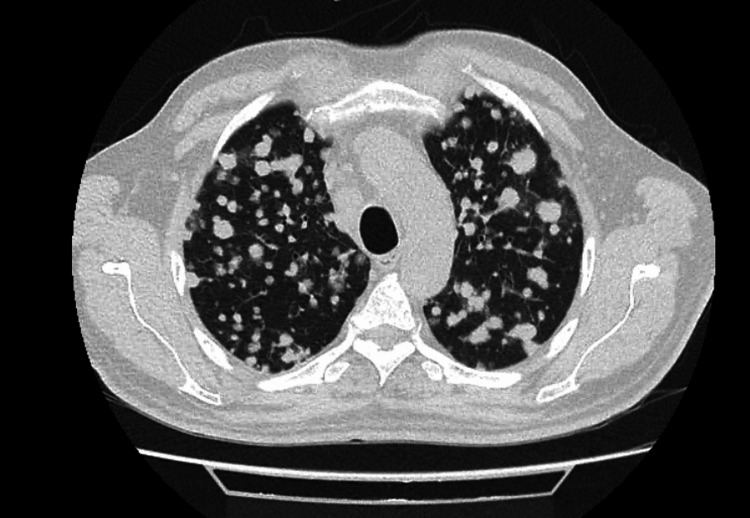
Chest CT scan revealing massive pulmonary metastases (Case 2) Extensive pulmonary metastases displaying a “balloon-release” pattern.

**Figure 9:**
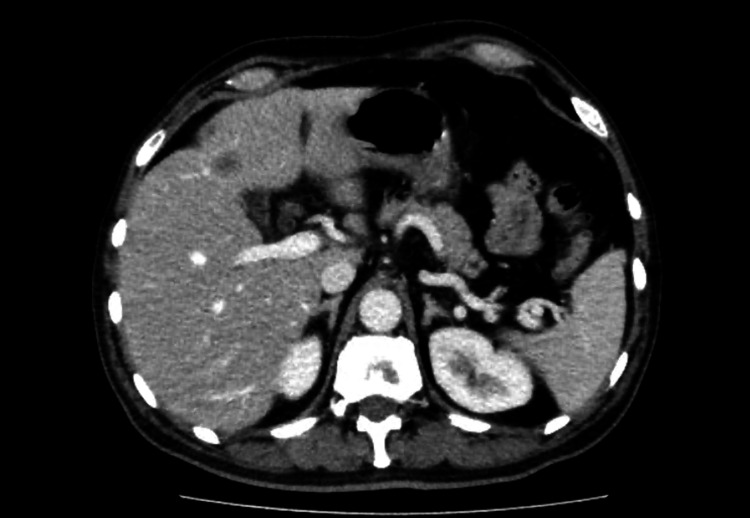
Abdominal CT scan showing new hepatic metastases (Case 2) De novo hepatic lesions.

However, two months later, the patient presented with worsening asthenia and hypoxemia (oxygen saturation of 81% on room air). Chest radiography revealed diffuse pulmonary nodular infiltrates, and CT confirmed massive pulmonary metastases along with new hepatic lesions (Figures 8, 9). Notably, PSA remained low at 0.04 ng/mL.

He was admitted for further evaluation and symptom management. A liver biopsy demonstrated large-cell neuroendocrine carcinoma, with immunohistochemical staining consistent with treatment-emergent neuroendocrine transformation. Due to rapid clinical deterioration, the patient died shortly thereafter.

## Discussion

Taken together, these two cases highlight the major clinical and biological challenges posed by NEPC. This entity represents an increasingly recognized mechanism of resistance in metastatic prostate adenocarcinoma, particularly in the era of potent ARPIs. Although still considered uncommon, its incidence appears to be rising, likely reflecting both selective pressure from prolonged androgen suppression and increasing diagnostic awareness. Both patients exemplified this phenomenon, with rapid and aggressive clinical deterioration.

In the first case, despite not meeting the CHAARTED (ChemoHormonal Therapy Versus Androgen Ablation Randomized Trial for Extensive Disease in Prostate Cancer) criteria for high-volume disease, the question arises as to whether early intensification with triplet therapy could have altered the disease trajectory [6]. Conversely, in the second case, neuroendocrine transformation appeared unavoidable, occurring despite initial treatment with docetaxel, darolutamide, and goserelin combination.

Another area of uncertainty concerns the role of local radiotherapy. Although emerging evidence suggests potential benefit in selected patients with metastatic disease, it remains unclear whether earlier prostate-directed radiotherapy might have impacted the course of either case [7,8].

A key clinical observation in both patients was radiologic progression in the context of disproportionately low PSA levels. This biochemical-radiologic discordance should raise suspicion for lineage plasticity and neuroendocrine differentiation. Current evidence supports early acquisition of new tumour tissue for histologic confirmation in such scenarios, particularly when clinical or imaging progression occurs without PSA elevation; this may be achieved through biopsy of an accessible metastatic site, as represented in both cases. Functional imaging, such as PSMA PET, may also show reduced uptake in NEPC, further suggesting dedifferentiation; in this setting, FDG PET is often more informative.

As illustrated in these cases, therapeutic options for NEPC remain limited and are largely extrapolated from treatment strategies for small-cell carcinomas of other origins, particularly the lung. Platinum-based chemotherapy remains the mainstay of treatment; however, responses are often short-lived, and overall survival remains poor. In Case 1, the platinum-taxane combination provided minimal benefit, underscoring the aggressive natural history of this disease subtype. There is an urgent need for dedicated clinical trials and predictive biomarkers to better guide treatment selection.

Recent advances have highlighted delta-like ligand 3 (DLL3) as a promising diagnostic and therapeutic target in NEPC (and similarly in small-cell lung cancer). Early clinical data have shown that DLL3-directed imaging agents can reliably detect DLL3-expressing lesions, as demonstrated in a 2024 pilot study [9]. In addition, emerging therapeutic approaches such as tarlatamab, a bispecific T-cell engager that redirects cytotoxic T cells toward DLL3-positive cells, have also yielded encouraging results [10].

Molecular profiling may occasionally reveal actionable genomic alterations, although clinically meaningful targets remain uncommon. In our first case, NGS identified a complete *MLH1* gene deletion, theoretically raising the possibility of benefit from PARP inhibitor therapy in combination with ARPI, due to replication stress and impaired DNA damage signalling, as reflected in the TALAPRO-2 trial [11,12]. Nevertheless, the clinical benefit of such approaches in NEPC remains unclear, and access to these therapies may be limited. This case illustrates the biological heterogeneity of NEPC and the challenges of translating genomic findings into effective treatment strategies in routine clinical practice.

These cases also reinforce the importance of early involvement of palliative care, given the substantial symptom burden and poor prognosis associated with NEPC.

## Conclusions

These two cases illustrate the clinical complexity and biological aggressiveness of NEPC. Despite advances in systemic therapy for metastatic prostate adenocarcinoma, NEPC remains a lethal resistance phenotype with limited therapeutic options and poor outcomes. Early recognition, particularly in the setting of radiologic progression with disproportionately low PSA, is essential, and timely histologic confirmation plays a central role in establishing the diagnosis. Although molecular profiling may occasionally identify genomic alterations of potential interest, the therapeutic implications of these findings (such as *MLH1* deletion) remain uncertain and should be interpreted with caution. Ultimately, these cases underscore the urgent need for improved diagnostic tools, novel therapeutic approaches, and dedicated clinical research aimed at better understanding and managing this increasingly encountered and highly aggressive disease.
